# Simultaneous multislice cardiac multimapping based on locally low-rank and sparsity constraints

**DOI:** 10.1016/j.jocmr.2024.101125

**Published:** 2024-11-14

**Authors:** Yixin Emu, Yinyin Chen, Zhuo Chen, Juan Gao, Jianmin Yuan, Hongfei Lu, Hang Jin, Chenxi Hu

**Affiliations:** a National Engineering Research Center of Advanced Magnetic Resonance Technologies for Diagnosis and Therapy, School of Biomedical Engineering, Shanghai Jiao Tong University, Shanghai, China; b Central Research Institute, UIH Group, Shanghai, China; c Department of Radiology, Zhongshan Hospital, Fudan University and Shanghai Medical Imaging Institute, Shanghai, China

**Keywords:** Simultaneous multislice, Parametric mapping, Multimapping, LLR, Compressed sensing

## Abstract

**Background:**

Although quantitative myocardial T1 and T2 mappings are clinically used to evaluate myocardial diseases, their application needs a minimum of six breath-holds to cover three short-axis slices. The purpose of this work is to simultaneously quantify multislice myocardial T1 and T2 across three short-axis slices in one breath-hold by combining simultaneous multislice (SMS) with multimapping.

**Methods:**

An SMS-Multimapping sequence with multiband radiofrequency (RF) excitations and Cartesian fast low-angle shot readouts was developed for data acquisition. When 3 slices are simultaneously acquired, the acceleration rate is around 12-fold, causing a highly ill-conditioned reconstruction problem. To mitigate image artifacts and noise caused by the ill-conditioning, a reconstruction algorithm based on locally low-rank and sparsity (LLRS) constraints was developed. Validation was performed in phantoms and in vivo imaging, with 20 healthy subjects and 4 patients, regarding regional mean, precision, and scan-rescan reproducibility.

**Results:**

The phantom imaging shows that SMS-Multimapping with locally low-rank (LLRS) accurately reconstructed multislice T1 and T2 maps despite a six-fold acceleration of scan time. Healthy subject imaging shows that the proposed LLRS algorithm substantially improved image quality relative to split slice-generalized autocalibrating partially parallel acquisition. Compared with modified look-locker inversion recovery (MOLLI), SMS-Multimapping exhibited higher T1 mean (1118 ± 43 ms vs 1190 ± 49 ms, P < 0.01), lower precision (67 ± 17 ms vs 90 ± 17 ms, P < 0.01), and acceptable scan-rescan reproducibility measured by 2 scans 10-min apart (bias = 1.4 ms for MOLLI and 9.0 ms for SMS-Multimapping). Compared with balanced steady-state free precession (bSSFP) T2 mapping, SMS-Multimapping exhibited similar T2 mean (43.5 ± 3.3 ms vs 43.0 ± 3.5 ms, P = 0.64), similar precision (4.9 ± 2.1 ms vs 5.1 ± 1.0 ms, P = 0.93), and acceptable scan-rescan reproducibility (bias = 0.13 ms for bSSFP T2 mapping and 0.55 ms for SMS-Multimapping). In patients, SMS-Multimapping clearly showed the abnormality in a similar fashion as the reference methods despite using only one breath-hold.

**Conclusion:**

SMS-Multimapping with the proposed LLRS reconstruction can measure multislice T1 and T2 maps in one breath-hold with good accuracy, reasonable precision, and acceptable reproducibility, achieving a six-fold reduction of scan time and an improvement of patient comfort.

## Graphical abstract

**Figure:**
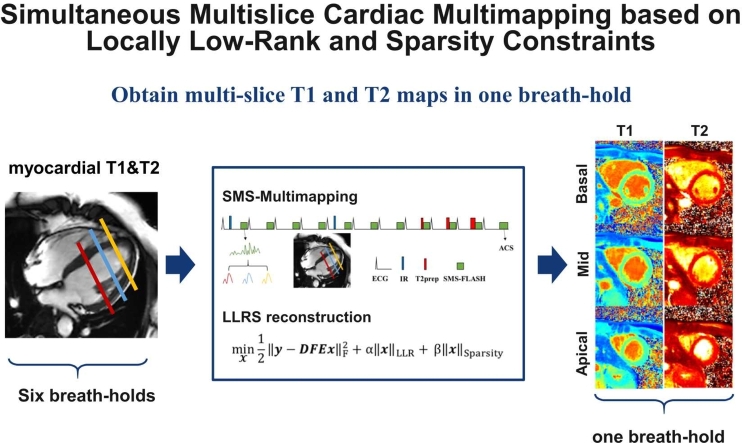

## 1 Background

Myocardial T1 and T2 mappings have been clinically used to evaluate fibrosis and edema, due to the sensitivity of T1 and T2 values to these myocardial abnormalities [1], [2], [3], [4]. Among them, myocardial T1 is often measured by the modified look-locker inversion recovery (MOLLI) sequence [4], while myocardial T2 is often measured by the T2-preparation balanced steady-state free precession (T2bSSFP) sequence [2]. Typically, these mapping sequences need to be performed in three short-axis slices and two long-axis slices [5]. Since each slice needs a single breath-hold, acquiring both T1 and T2 mapping in three short-axis slices needs a minimum of six separate breath-holds. These repeated breath-holds not only cause a long examination time, but also increase the chance of patient discomfort and motion artifacts. Therefore, accelerating multislice multiparametric mapping is desirable from a clinical perspective.

To date, several strategies have been developed to accelerate multislice multiparametric mapping. One strategy is to obtain T1 and T2 mapping data in a single scan. Methods using this strategy include magnetic resonance fingerprinting (MRF) [6], multitasking [7], multimapping [8], and several joint T1-T2 mapping methods [9], [10]. A common feature shared by these methods is the inclusion of multiple inversion recovery models and T2 preparations in the sequence to generate different T1 and T2 signal weightings. Among these methods, cardiac multimapping [8] combines Cartesian k-space sampling with dictionary matching and fulfills joint T1 and T2 mapping in 10 heartbeats, which is a reasonable duration for breath-holding. However, for these methods, acceleration is only along the parametric dimension; three separate breath-holds are still needed to measure myocardial T1 and T2 in 3 short-axis slices.

To accelerate imaging along the slice direction, simultaneous multislice (SMS) [11] can be used, which is the second strategy for the acceleration of parametric mapping. To use SMS, a multiband (MB) radiofrequency (RF) pulse needs to be used, which is different depending on whether fast low-angle shot (FLASH) [12], [13], [14], [15], [16], [17], [18] or bSSFP [19], [20], [21] readouts are used. SMS has been applied to various parametric mapping tasks, including T1 mapping [14], [16], [18], [19], [20], T2 mapping [22], MRF [12], [13], [15], and multitasking [17]. Among them, SMS-MRF [15] and SMS-multitasking [17] were able to achieve multislice quantification of T1 and T2. Nevertheless, the SMS-MRF dictionary generation requires a substantially longer computational time than multimapping, while for SMS-multitasking, the unresolved motion due to free-breathing may induce blurring in the reconstructed maps [17]. Furthermore, both techniques entail longer scan time (a 16-heartbeat breath-hold or a 3-min free-breathing scan, respectively) than standard multimapping, raising challenges for routine clinical applications. The reconstruction for SMS multiparametric mapping also faces challenges. Since both in-plane and through-plane accelerations are needed for this application, the large undersampling rate can cause severe noise and artifacts in the reconstructed maps. A common reconstruction method for SMS imaging is split slice-generalized autocalibrating partial parallel acquisition ) (SPSG) [11], [12], [13], [14], [19], [22], [23], which has also been used in a recent work for SMS-T1 mapping [19]. However, whether it can achieve satisfactory performance in highly accelerated SMS multiparametric mapping remains unknown.

In this work, we sought to combine SMS with multimapping to achieve three-slice T1 and T2 mapping in one breath-hold. The original multimapping sequence was modified to use FLASH readouts equipped with SMS RF pulses. We show that SPSG caused dramatic noise and artifacts in the reconstruction, which was substantially reduced by the proposed reconstruction algorithm based on locally low-rank [18], [24], [25] and sparsity [25], [26] (LLRS) constraints. The proposed method was validated via phantom and in vivo imaging, for which both simulated and prospective accelerations were performed for the assessment.

## 2 Methods

### 2.1 Sequence design

**Fig. 1:**
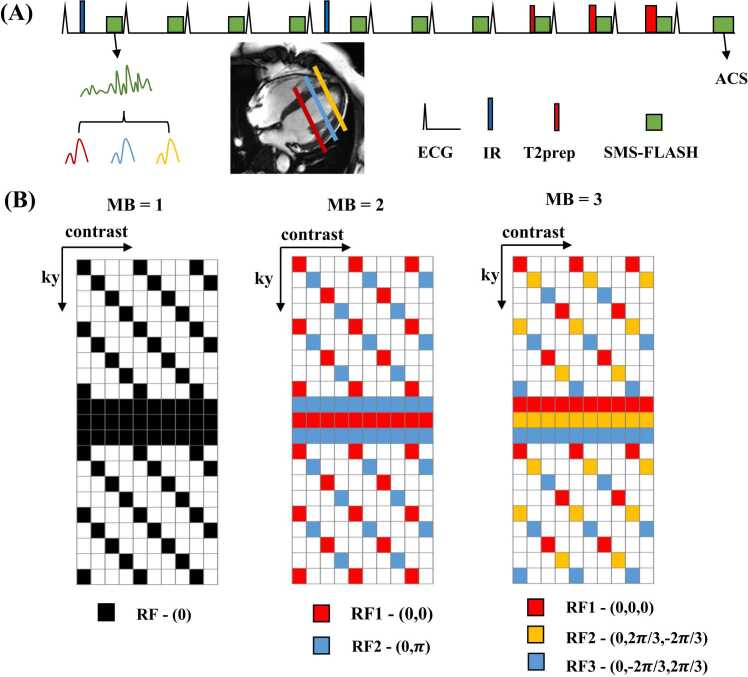
Schematic of the proposed sequence and the k-space sampling trajectory. (A) A schematic of the SMS-Multimapping sequence, which uses multiband RF pulses and FLASH readouts to acquire the SMS data. A heartbeat is added to obtain the full-sampled (18 centerlines) automatic calibration signal (ACS) for each slice. (B) The sequence samples k-space based on the recently proposed SUPER-CAIPIRINHA pattern. This pattern includes accelerations along both the in-plane and through-plane directions, among which the in-plane acceleration was shifted at every contrast. In this work, the total undersampling rates of k-space for simultaneous acquisition of 1, 2, and 3 slices are 4, 8, and 12, respectively. *SMS* simultaneous multislice, *RF* radiofrequency, *IR* inversion recovery, *T2prep* T2-preparation, *MB* multiband, *FLASH* fast low-angle shot, *ECG* electrocardiogram, *SUPER* shift undersampling improves parametric mapping efficiency and resolution, *CAIPIRINHA* controlled aliasing in parallel imaging results in higher acceleration, *RF* radiofrequency

Fig. 1A shows the design of the SMS-Multimapping sequence. The sequence uses the same scheme as multimapping [8] to generate different T1- and T2-weighted images in 10 heartbeats based on inversion recovery and T2-preparation. To accomplish SMS imaging, the sequence uses an MB RF excitation in combination with a Cartesian FLASH readout. Moreover, the sequence includes an additional heartbeat at the end of the sequence to obtain full-sampled (18 centerlines) automatic calibration signals for each slice, increasing the total acquisition time to 11 heartbeats. Fig. 1B shows a schematic of the k-space sampling trajectory, which is based on a previously published shift undersampling improves parametric mapping efficiency and resolution (SUPER)-controlled aliasing in parallel imaging results in higher acceleration (CAIPIRINHA) [27] undersampling pattern. This pattern is based on an internal shift undersampling (SUPER) of a CAIPIRINHA undersampling pattern, which in our case is a skewed grid in the kyz plane with a dense sampling of the central 24 k-space lines. Overall, the strategy leads to an undersampling of roughly four along the ky direction and an undersampling of one, two, or three along the kz direction, depending on the MB factor. The combined acceleration factor is thus 4, 8, and 12 for single-slice, 2-slice, and 3-slice SMS-Multimapping, respectively.

### 2.2 LLRS reconstruction

The acquired k-space data can be modeled as:

(1) $$\begin{array}{c} y\;=\mathrm{DFE}x+n \end{array}$$

where ***y*** is the acquired k-space data, ***D*** is the undersampling operator, ***F*** is the 3D Fourier transform, ***E*** is the coil sensitivity encoding matrix, ***x*** is the multislice multicontrast images to be estimated, and ***n*** is the noise. The cost function for the underlying reconstruction problem is then formulated as follows, which comprises the addition of three terms, namely the data fidelity term, the locally low-rank (LLR) constraint, and the sparsity constraint:

(2) $$\min_{x}\;\frac{1}{2}\|y-DFEx{\|}_{F}^{2}+\alpha \sum_{q=1}^{Q}\sum_{i=1}^{N_{v}}\|B_{i}x_{q}{\|}_{*}+\beta \sum_{q=1}^{Q}\|Wx_{q}{\|}_{1}$$

where $\|∙{\|}_{F}$ is the matrix Frobenius norm, $x_{q}$ is the *q*th slice of image, $B_{i}$ is the block-extraction operator, which takes a block of voxels centered at the ith voxel to form a Casorati matrix, $\|∙{\|}_{*}$ is the nucleus norm, W is the wavelet transform, ${\left\|∙\right\|}_{1}$ is the $l_{1}$-norm, $N_{v}$ is the number of voxels, Q is the number of slices (MB factor), and α and β are the weights of the LLR and sparsity constraints, respectively. Note that both LLR and sparsity constraints are separately applied to each slice.

The above nonlinear inverse problem is solved by the alternating direction method of multipliers (ADMM) [28] algorithm. To proceed, we reformulated Eq. (2) as

(3) $$\min_{x}\;\frac{1}{2}\|y-DFEx{\|}_{F}^{2}+\alpha \sum_{q=1}^{Q}\sum_{i=1}^{N_{v}}\|z_{iq}{\|}_{*}+\beta \sum_{q=1}^{Q}\|v_{q}{\|}_{1}$$

$$s.t.\;B_{i}x_{q}=z_{iq},\;Wx_{q}=v_{q}\;$$

which is then followed by forming the augmented Lagrangian function:

(4) minx,{zi},v12‖y-DFEx‖F2+α∑q=1Q∑i=1Nv‖ziq‖*+β∑q=1Q‖vq‖1+∑q=1Q∑i=1NvReλiq,Bixq−ziq+μ12∑q=1Q∑i=1Nv‖Bixq−ziq‖F2+∑q=1QReζq,Wxq−vq+μ22∑q=1Q‖Wxq−vq‖F2

where $\lambda_{\mathrm{iq}}\;$ and $\zeta_{q}$ are the Lagrange multipliers corresponding to the first and second constraint, respectively, and $\mu_{1}$ and $\mu_{2}$ are the weights of the augmented penalty terms. The ADMM algorithm then alternatingly minimizes the above function with respect to each unknown variable by executing the following four steps in the kth iteration:

$\mathrm{Step}1:x^{k+1}={\mathrm{argmin}}_{x}\;\frac{1}{2}\|y-DFEx{\|}_{F}^{2}+\frac{\mu_{1}}{2}\overset{Q}{\underset{q=1}{\sum }}\overset{N_{v}}{\underset{i=1}{\sum }}\|B_{i}x_{q}-(z_{iq}^{k}-\frac{\lambda_{iq}^{k}}{\mu_{1}}){\|}_{F}^{2}+\frac{\mu_{2}}{2}\overset{Q}{\underset{q=1}{\sum }}\|Wx_{q}-(v_{q}^{k}-\frac{\zeta_{q}^{k}}{\mu_{2}}){\|}_{F}^{2}$

for each q.

$\mathrm{Step}3:v_{q}^{k+1}={\mathrm{argmin}}_{v_{q}}\beta {\left\|v_{q}\right\|}_{1}+\frac{\mu_{2}}{2}{\left\|Wx_{q}^{k+1}-(v_{q}-\frac{\zeta_{q}^{k}}{\mu_{2}})\right\|}_{F}^{2}$ for each q.

$\mathrm{Step}4:\lambda_{\mathrm{iq}}^{k+1}=\lambda_{\mathrm{iq}}^{k}+\mu_{1}(B_{i}x_{q}^{k+1}-z_{\mathrm{iq}}^{k+1})$, $\zeta_{q}^{k+1}=\zeta_{q}^{k}+\mu_{2}(Wx_{q}^{k+1}-v_{q}^{k+1})$ for each q.

where $x_{q}^{k},z_{\mathrm{iq}}^{k},\;v_{q}^{k},\;\lambda_{\mathrm{iq}}^{k}$ and $\zeta_{q}^{k}$ are the kth iterate of $x_{q},z_{\mathrm{iq}},\;v_{q},\;\lambda_{\mathrm{iq}}$ and $\zeta_{q}$, respectively. The pseudocode of the LLRS algorithm is shown in Table 1. Note that unlike SPSG, SMS unfolding is not separately performed in our reconstruction because we model the whole reconstruction as a three-dimensional dynamic image reconstruction problem and solve it using the LLRS algorithm.

**Table 1:** Pseudocode of the LLRS algorithm for SMS-Multimapping.

ADMM algorithm for LLRS
Optional parameters: MaxIter-maximal number of iterations (default = 100)
% Initialization
$k=0\text{; }\lambda_{\mathrm{iq}}^{0}=0\text{; }\zeta_{q}^{0}=0\text{; }z_{\mathrm{iq}}^{0}=0;\;v_{q}^{0}=0$

% Step 1
$x^{k+1}={\left(E^{H}F^{H}D^{H}\mathrm{DFE}+\mu_{1}\sum_{i=1}^{N_{v}}B_{i}^{H}B_{i}+\mu_{2}W^{H}W\right)}^{-1}$
$\left(E^{H}F^{H}D^{H}y+\mu_{1}\sum_{q=1}^{Q}\sum_{i=1}^{N_{v}}B_{i}^{H}(z_{iq}^{k}-\frac{\lambda_{iq}^{k}}{\mu_{1}})+\mu_{2}\sum_{q=1}^{Q}W^{H}(v_{q}^{k}-\frac{\zeta_{q}^{k}}{\mu_{2}})\right)$

% Step 2
$z_{\mathrm{iq}}^{k+1}={\mathrm{SVT}}_{\frac{\alpha }{\mu_{1}}}\left(B_{i}x_{q}^{k+1}+\frac{\lambda_{\mathrm{iq}}^{k}}{\mu_{1}}\right)$

% Step 3
$v_{q}^{k+1}={\mathrm{ST}}_{\frac{\beta }{\mu_{2}}}\;\left(Wx_{q}^{k+1}+\frac{\zeta_{q}^{k}}{\mu_{2}}\right)$

% Step 4
$\lambda_{\mathrm{iq}}^{k+1}=\lambda_{iq}^{k}+\mu_{1}\left(B_{i}x_{q}^{k+1}-z_{\mathrm{iq}}^{k+1}\right)$
$\zeta_{q}^{k+1}=\zeta_{q}^{k}+\mu_{2}\left(Wx_{q}^{k+1}-v_{q}^{k+1}\right)$

% Step 5
k=k+1

*LLRS* locally low-rank and sparsity, *SMS* simultaneous multislice, *ADMM* alternating direction method of multipliers

SVT singular value thresholding, ST soft thresholding, Step 1 is separately implemented at each point along the readout direction. Steps 2-4: for each q.

### 2.3 Dictionary matching

Once the raw images are reconstructed, dictionary matching is used to estimate the multislice T1 and T2 maps from the raw images. The dictionary is generated by Bloch equation simulation of the SMS-Multimapping sequence. The inversion efficiency is assumed to be 0.94 in the phantom and in vivo experiment [8], [29]. Following the original work of multimapping [8], the B1 field is first estimated using a relatively abbreviated dictionary that involves T1 (range: [500 ms:100 ms:1500 ms]), T2 (range: [40 ms:30 ms:140 ms]), and B1 (range: [0.5:0.05:1]). Then, the mean B1 in the septum is used as an input for constructing a highly resolved dictionary over variations of T1 and T2 to facilitate accurate mapping. The highly resolved dictionary for phantom imaging had a T1 range of [1 ms:10 ms:2500 ms] and a T2 range of [1 ms:4 ms:400 ms]. The highly resolved dictionary for in vivo imaging had a T1 range of [200 ms:1 ms:2500 ms] and T2 range of [1 ms:1 ms:150 ms].

### 2.4 Data acquisition

All magnetic resonance imaging experiments were performed in 3T scanners (uMR790, Shanghai United Imaging Healthcare, Shanghai, China) with a 24-channel spine coil and 12-channel torso coil.

#### 2.4.1 Phantom imaging

We measured the slice profile for the proposed SMS-Multimapping method,by adding a readout gradient in the slice direction following the SMS RF pulse and performed the imaging in a homogeneous doped-water phantom [19]. Typical acquisition parameters of the slice profile sequence included: field of view (FOV) = 300 × 300 mm^2^, matrix size = 256 × 256, flip angle = 5°, bandwidth = 400 Hz/pixel, and slice thickness = 8 mm. We measured the slice profile for MB = 2 and 3 with a variety of interslice gaps.

We validated the accuracy of SMS-Multimapping with two different phantoms. In the first experiment, we used the International Society for Magnetic Resonance in Medicine/National Institute of Standards and Technology (ISMRM/NIST) phantom [30] and measured its multislice T1 and T2 with the proposed approach for a range of simulated heart rates. The purpose of this experiment was to validate whether SMS-Multimapping with the LLRS reconstruction can produce high-quality T1 and T2 maps. However, since the ISMRM/NIST phantom does not use T1s and T2s that match those of the myocardium, we repeated the experiment in another phantom that contained nine vials filled with mixed solutions of NiCl_2_ and agar gel. A number of simulated heart rates from 40 to 120 bpm were used to test the reproducibility of the proposed method with heart rate variations. Typical acquisition parameters of the sequence included: FOV = 360 × 270 mm^2^, matrix size = 192 × 138, flip angle = 5°, repetition time (TR)/echo time (TE) = 4.19 ms/1.73 ms, bandwidth = 400 Hz/pixel, and slice thickness = 8 mm. Reference T1 and T2 mapping was performed in the middle slice using T1-weighted (13TIs, from 100 to 9500 ms) and T2-weighted (9TEs, from 15 to 300 ms) spin echo (SE) sequences.

#### 2.4.2 In vivo imaging

The study was approved by the institutional review board from each participating institution, and written informed consent was provided by every subject and patient before the scan.

In vivo imaging was first performed in 3 short-axis slices of 20 healthy subjects (10 males, age 23 ± 3 years). Acquisition parameters for healthy subjects were the same with those for the phantom study. The distance between adjacent slices was 12.8 mm. To evaluate the proposed method, MOLLI, bSSFP T2 mapping, and multimapping were acquired separately in the same slices with the same acquisition parameters except: flip angle = 35°, TR/TE = 2.8 ms/1.3 ms, bandwidth = 1200 Hz/pixel, and generalized autocalibrating partially parallel acquisition (GRAPPA) [31] factor = 2. To assess the scan-rescan reproducibility, we repeated the scan after 10 min in 10 healthy subjects.

In vivo imaging was also performed on four patients (1 male, age 42 ± 13 years) at Zhongshan Hospital, Shanghai, China. SMS-Multimapping and clinical imaging sequences (Cine, MOLLI, T2 mapping, and late gadolinium enhancement [LGE]) were performed during breath-hold scans. The acquisition parameters for SMS-Multimapping, MOLLI, and T2 mapping were the same as those for healthy subjects except: FOV = 360 × 320 mm^2^ and matrix size = 192 × 145. The slice gap varied between 12 and 20 mm, depending on the patient’s heart size. Clinical indications included myocardial infarction (n = 1), heart failure (n = 1), hypertrophic cardiomyopathy (n = 1), and myocarditis (n = 1).

### 2.5 Details of image reconstruction

Eigenvalue iTerative Self-consistent Parallel Imaging Reconstruction (ESPIRiT) [32] was used to estimate coil sensitivity maps for all parameter mapping methods. The proposed LLRS algorithm for the prospective SMS-Multimapping reconstruction used the following parameters: block size = 7, $\alpha =10^{-3.75}$, $\mu_{1}=10^{-2.75},\;\beta =10^{-4},\;\mu_{2}=10^{-3}$. A maximal number of 100 iterations was used for LLRS based on observations. With these settings, it took about 30 min for LLRS to reconstruct a three-slice SMS-Multimapping dataset. After LLRS reconstruction, a registration method (pTVreg) [33] was performed with default parameter values to correct motion in the patients. Phase-sensitive correction was then performed on the raw images to determine the polarity of the signal [8], [34], followed by dictionary matching. All reconstructions were performed with MATLAB (The MathWorks, Natick, Massachusetts) on a computational server equipped with an 8-core i7-9700K CPU (Intel, Santa Clara, California).

### 2.6 Image analysis

To evaluate the proposed method, five experiments were performed. Experiment 1 investigated the accuracy and heart rate dependency of the proposed method by phantom imaging. Experiment 2 performed undersampling after simulating the SMS k-space data using reconstructed single-slice data (MB = 1) and compared LLRS with SPSG+GRAPPA for reconstruction of the simulated SMS k-space data. This comparison was performed on randomly chosen 10 subjects. White Gaussian noise with a standard deviation (SD) of 0.005 was added to the simulated SMS k-space data. Experiment 3 compared LLRS with SPSG+GRAPPA using the prospectively undersampled SMS data. Experiment 4 compared the accuracy, precision, and reproducibility of the proposed method with MOLLI, bSSFP T2 mapping, and multimapping. Experiment 5 investigated the qualitative scoring of each imaging method. The patient data were visually examined to preliminarily evaluate the performance of the proposed method in the clinical setting.

For phantom study, maps were analyzed by drawing regions of interest (ROI) within each vial in phantom 2 and computing the mean and SD. Correlation coefficients and Bland-Altman analyses were performed to evaluate the quantitative agreement between SMS-Multimapping and reference methods. For the simulation study, peak signal-to-noise ratio (PSNR) and the structural similarity index measure (SSIM) were evaluated for each method over all subjects, all slices, and all raw images. For prospective in vivo imaging, the myocardium in T1 and T2 maps was segmented according to the 16 AHA segment model [35], and the segmental mean and SD were analyzed. Bland-Altman analysis was conducted to evaluate the reproducibility of each method.

Qualitative scoring was performed to evaluate the performance of each mapping method. Two experienced readers (Z.C. and J.G., both with more than 3 years of cardiovascular magnetic resonance) independently and blindly reviewed the maps of all slices. Three criteria, namely the artifacts, sharpness of myocardial boundaries, and overall image quality, were graded with a 5-point scale (1: nondiagnostic; 2: poor; 3: fair; 4: good; and 5: excellent). The scores from the two readers were averaged to obtain the final score.

### 2.7 Statistical analysis

Bland-Altman plots were used to evaluate the T1 and T2 differences between the different mapping methods and different scans. One-way analysis of variance and post hoc test were used to assess the differences in quantitative parameters between different imaging methods. The Kruskal-Wallis test was used to evaluate differences between qualitative comparisons. The Bonferroni correction was applied as a P-value adjustment when multiple comparisons were made. The inter-observer agreement was evaluated by intraclass correlation coefficient (ICC). P < 0.05 was considered statistically significant. Statistical analyses were performed using IBM SPSS Statistics (version 27.0, IBM, Armonk, New York, USA).

## 3 Results

### 3.1 Phantom imaging

**Fig. 2:**
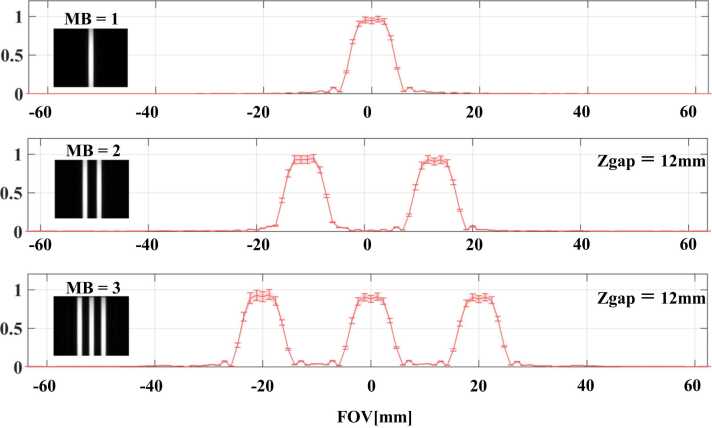
Slice profile for different MB factors and a fixed slice gap (Zgap) of 12 mm. *MB* multiband, *FOV* field of view

Fig. 2 shows the slice profile of the SMS pulse for different MB factors and a fixed slice gap (Zgap) of 12 mm. Fig. S1 shows additional measurements for different slice gaps at MB = 3. The results show that an interslice gap of 12 mm was sufficient to minimize cross-talk between slices.

**Fig. 3:**
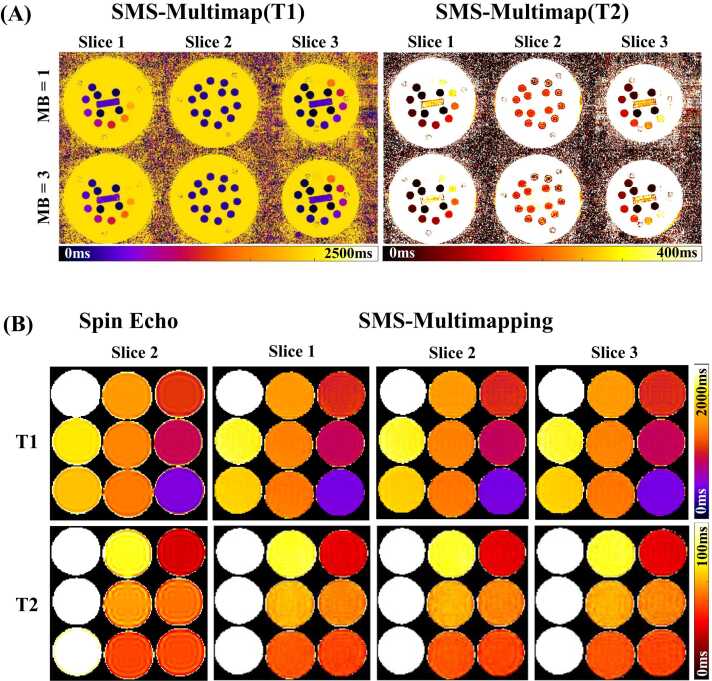
T1 and T2 maps for phantoms obtained by SMS-Multimapping and reference. (A) The reconstructed ISMRM/NIST phantom SMS-multimaps for multiband (MB) factors of 1 and 3 at an 80 bpm heart rate. The T1 and T2 maps at MB = 3 were in good agreement with those acquired slice-by-slice (MB = 1). (B) The spin-echo-measured maps of the center slice and the SMS-multimaps (MB = 3) of three slices of the nine-vial phantom. The T1 and T2 values measured by SMS-Multimapping in each vial were visually in good agreement with those measured by spin echo. *SMS* simultaneous multislice, *ISMRM/NIST*, International Society for Magnetic Resonance in Medicine/National Institute of Standards and Technology

Fig. 3A shows the SMS-multimaps of the ISMRM/NIST phantom for MB factors 1 and 3. Visual inspection revealed that the proposed method satisfactorily disentangled different slices, generating maps of consistent image qualities between the two MB factors. Fig. 3B shows the SE-measured maps and SMS-multimaps for the other phantom, which has physiologically relevant T1 and T2 values. The T1s and T2s measured by SMS-Multimapping are visually in good agreement with those measured by SE.

**Fig. 4:**
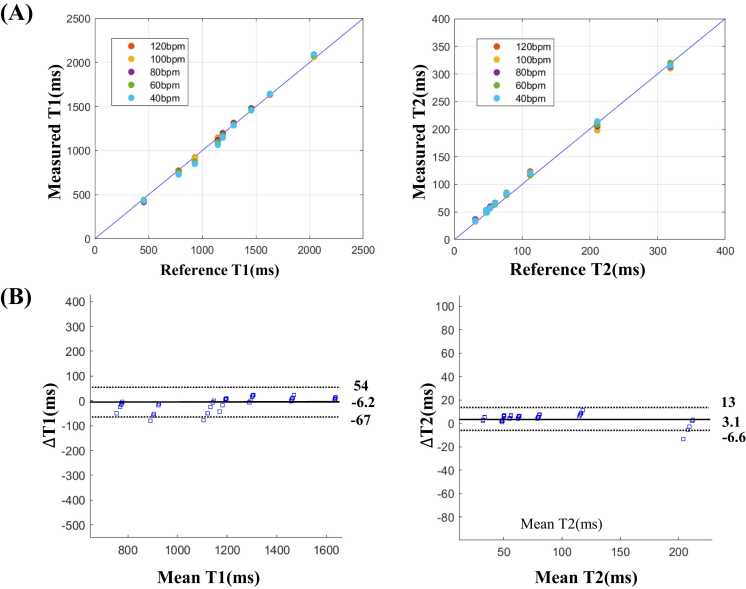
T1 and T2 values measured by SMS-Multimapping (MB = 3) in the nine-vial phantom for different heart rates from 40 to 120 bpm, compared to reference values. (A) T1 and T2 values measured by SMS-Multimapping well correlated with those measured by spin echo imaging across different heart rates (R^2^ > 0.99). (B) Bland-Altman analyses show that the mean difference between SMS-Multimapping (MB = 3) and spin echo imaging was −6.2 ms for T1 and 3.1 ms for T2. *SMS* simultaneous multislice, *MB* multiband

Fig. 4A shows the comparison of average T1 and T2 values measured by SMS-Multimapping and those measured by SE imaging for different heart rates. R^2^ was above 0.99 between the estimated T1 or T2 and the reference values across all heart rates. Bland-Altman analyses (Fig. 4B) show that the bias for T1 measurement was −6.2 ms (upper 95% limits of agreement = 54 ms, lower 95% limits of agreement = −67 ms), and for T2 was 3.1 ms (upper 95% limits of agreement = 13 ms, lower 95% limits of agreement = −6.6 ms).

### 3.2 Simulated in vivo imaging

**Fig. 5:**
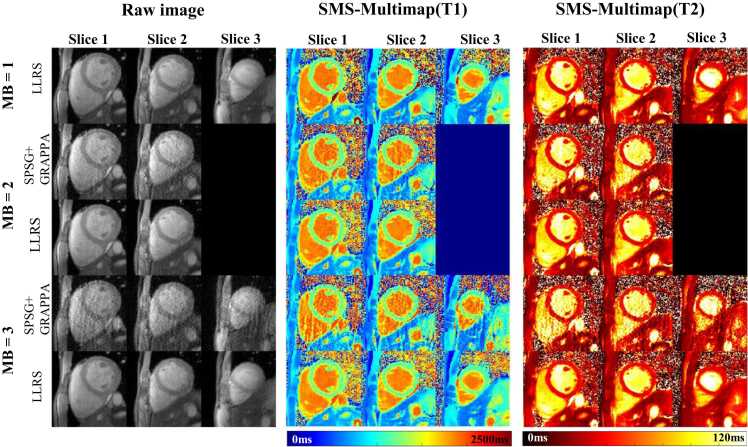
Reconstructions of simulated in vivo SMS-Multimapping by SPSG+GRAPPA and the proposed LLRS method. For MB = 2 and 3, the traditional reconstruction method SPSG+GRAPPA manifested strong aliasing artifacts and noise amplification both in the raw images and maps. The quality of LLRS (MB = 2/3) was overall agreeable with the ground truth (MB = 1). *MB* multiband, *SPSG* split slice-GRAPPA, *LLRS* locally low-rank and sparsity, *SMS* simultaneous multislice, *GRAPPA* generalized autocalibrating partially parallel acquisition

Fig. 5 shows the reconstructed raw images in the first heartbeat and the SMS-multimaps with MB = 1, 2, and 3, reconstructed with SPSG+GRAPPA and LLRS, based on k-space data simulated from the MB = 1 images (ground truth). SPSG+GRAPPA exhibited visible aliasing artifacts in the raw images and maps. On the contrary, LLRS exhibited considerably fewer aliasing artifacts compared with SPSG+GRAPPA and maintained a similar image quality relative to the ground truth. Table 2 shows the PSNR and SSIM (mean ± SD) of the raw image reconstruction over 10 subjects for MB of 2 and 3. LLRS showed significantly higher PSNR and SSIM by a large margin compared with SPSG+GRAPPA (P < 0.01 for all comparisons).

**Table 2:** PSNR/SSIM of LLRS and SPSG+GRAPPA over 10 healthy subjects.

MB factor	Method	PSNR	SSIM
MB = 2	GRAPPA+SPSG	21.23 ± 0.87	78.05 ± 3.05
	LLRS	40.59 ± 0.73	96.76 ± 0.16
MB = 3	GRAPPA+SPSG	18.19 ± 0.97	69.04 ± 2.39
	LLRS	38.99 ± 0.89	96.01 ± 0.25

Data presented as mean ± standard deviation. *PSNR* peak signal-to-noise ratio, *SSIM* structural similarity index measure, *LLRS* locally low-rank and sparsity, *GRAPPA* generalized autocalibrating partially parallel acquisition, *SPSG* split slice-GRAPPA, *MB* multiband

### 3.3 Prospective in vivo imaging

**Fig. 6:**
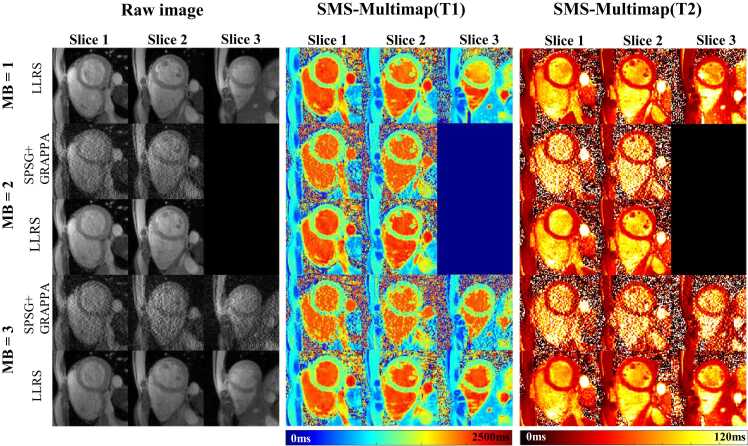
Reconstructions of prospectively accelerated in vivo SMS-Multimapping by SPSG+GRAPPA and LLRS for MB = 2 and 3. For MB = 1, reconstruction based on LLRS was also shown. LLRS substantially reduced noise and aliasing artifacts compared with SPSG+GRAPPA. The quality of LLRS at MB = 2 well agreed with that of LLRS at MB = 1. The quality of LLRS at MB = 3 was overall agreeable with that of LLRS at MB = 1, although some blurring was visible around small structures. *MB* multiband, *SPSG* split slice-GRAPPA, *LLRS* locally low-rank and sparsity, *GRAPPA* generalized autocalibrating partially parallel acquisition, *SMS* simultaneous multislice

Fig. 6 shows the reconstruction results of prospectively accelerated in vivo SMS-Multimapping by SPSG+GRAPPA and LLRS for MB = 2 and 3. The reconstruction at MB = 1 based on LLRS was also shown. Consistent with the simulation results, LLRS substantially reduced noise and aliasing artifacts compared with SPSG+GRAPPA. The quality of LLRS at MB = 3 was overall agreeable with that of LLRS at MB = 1. However, some blurring can be observed when MB = 3, mainly for small structures such as the papillary muscles and trabeculations.

**Fig. 7:**
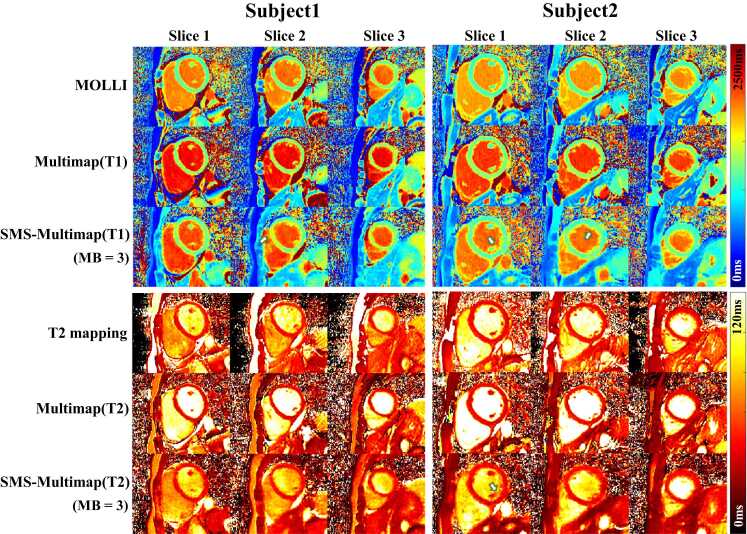
T1 and T2 maps obtained by SMS-Multimapping for MB = 3 (one breath-hold), and their comparison with the reference standards, including MOLLI and T2 mapping (six breath-holds), multimapping (three breath-holds) in two subjects. Despite the six-fold acceleration, SMS-Multimapping exhibited an overall similar quality compared with MOLLI and T2 mapping. Some blurring (white arrows) can be observed in SMS-Multimapping due to the high acceleration rate. *SMS* simultaneous multislice, *MB* multiband, *MOLLI* modified look-locker inversion recovery

Fig. 7 shows the T1 and T2 maps obtained by SMS-Multimapping for MB = 3 (three breath-hold), and their comparison with the reference methods, including MOLLI and T2 mapping (six breath-holds), and multimapping (three breath-holds) in two subjects. Despite the six-fold acceleration, SMS-Multimapping exhibited an overall similar quality compared with MOLLI and T2 mapping. Some blurring (white arrows) can be observed in SMS-Multimapping. However, note that the imaging time was reduced by a factor of 6 over standard mapping and 3 over multimapping.

**Fig. 8:**
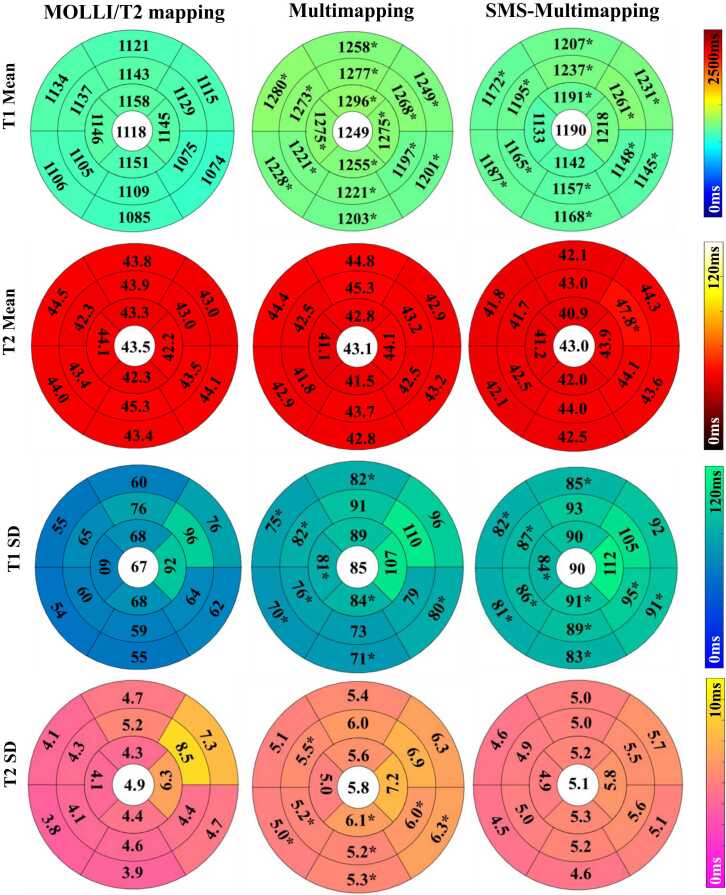
Comparison of regional T1/T2 mean and SD between MOLLI/T2 mapping, multimapping, and SMS-Multimapping (MB = 3) over 20 subjects. Both mutlimapping and SMS-mutlimapping showed higher regional T1s relative to MOLLI and similar regional T2s relative to T2 mapping. For T1, multimapping and SMS-Multimapping both had lower precisions than MOLLI. For T2, the precision of SMS-Multimapping appeared to be superior to multimapping and similar to T2 mapping. The symbol * represents P < 0.05 compared to MOLLI/T2 mapping. *SD* standard deviation, *MOLLI* modified look-locker inversion recovery, *MB* multiband, *SMS* simultaneous multislice

Fig. 8 shows the comparisons between MOLLI/T2 mapping, multimapping, and SMS-Multimapping (MB = 3) in terms of regional T1/T2 means and SDs over 20 subjects. Three methods had significant differences for T1 means (P < 0.01), SDs (P < 0.01), and T2 SDs (P = 0.02). Both mutlimapping (1249 ± 49 ms) and SMS-mutlimapping (1190 ± 49 ms) showed higher regional T1s relative to MOLLI (1118 ± 43 ms; P < 0.01 for both), due to the use of dictionary matching. On the other hand, their T2 means (43.5 ± 3.3 ms for T2 mapping, 43.1 ± 4.1 ms for multimapping, 43.0 ± 3.5 ms for SMS-mutlimapping) were similar (P = 0.43). There was no significant difference between T2 mapping and SMS-mutlimapping (P = 0.64). The T1 precision of SMS-Multimapping (90 ± 17 ms) was similar to multimapping (84 ± 20 ms, P = 0.34), and both of them were lower than the precision of MOLLI (67 ± 17 ms; P < 0.01 for both). On the other hand, the T2 precision of SMS-Multimapping (5.1 ± 1.0 ms) was superior to multimapping (5.8 ± 1.3 ms; P = 0.03) and similar to T2 mapping (4.9 ± 2.1 ms; P = 0.93). Note that the accuracy and precision were overall similar between multimapping and SMS-Multimapping, despite a three-fold higher acceleration rate for the latter.

**Fig. 9:**
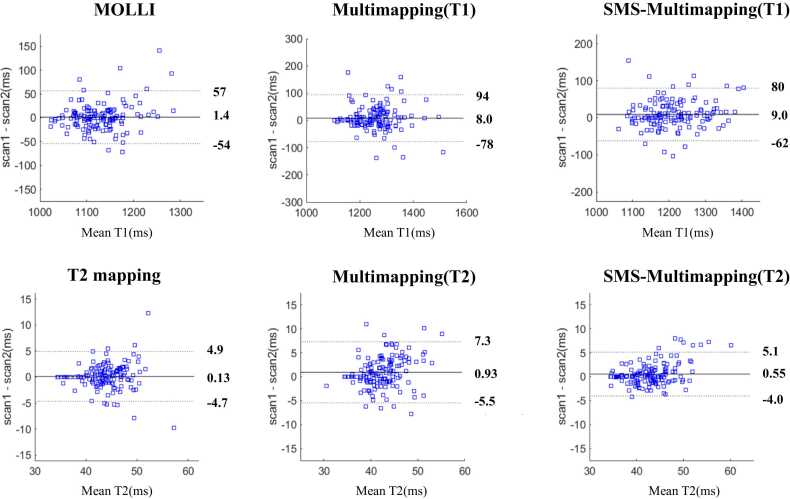
Assessment of scan-rescan reproducibility of MOLLI/T2 mapping, multimapping, and SMS-Multimapping (MB = 3) across 10 healthy subjects, where the two scans were separated by a 10-min interval. The reproducibility of SMS-Multimapping was similar to that of multimapping, and slightly worse than that of MOLLI. Considering the large T1 and T2 values of the myocardium, the reproducibility of SMS-Multimapping is in an acceptable range. *MOLLI* modified look-locker inversion recovery, *MB* multiband, *SMS* simultaneous multislice

Fig. 9 shows the Bland-Altman analysis of the scan-rescan difference for MOLLI/T2 mapping, multimapping, and SMS-Multimapping (MB = 3), with the two scans performed 10 min apart. The mean T1 bias between the two scans was 1.4 ms, 8.0 ms, and 9.0 ms for MOLLI, multimapping, and SMS-Multimapping, respectively. The mean T2 bias between the two scans was 0.13 ms, 0.93 ms, and 0.55 ms for T2 mapping, multimapping, and SMS-Multimapping, respectively. The results showed that the scan-rescan reproducibility was reduced for both multimapping and SMS-Multimapping compared with MOLLI and T2 mapping; however, no large difference was found between multimapping and SMS-Multimapping, and their T1 and T2 interscan differences were still acceptable considering the large T1 and T2 values of myocardium.

**Fig. 10:**
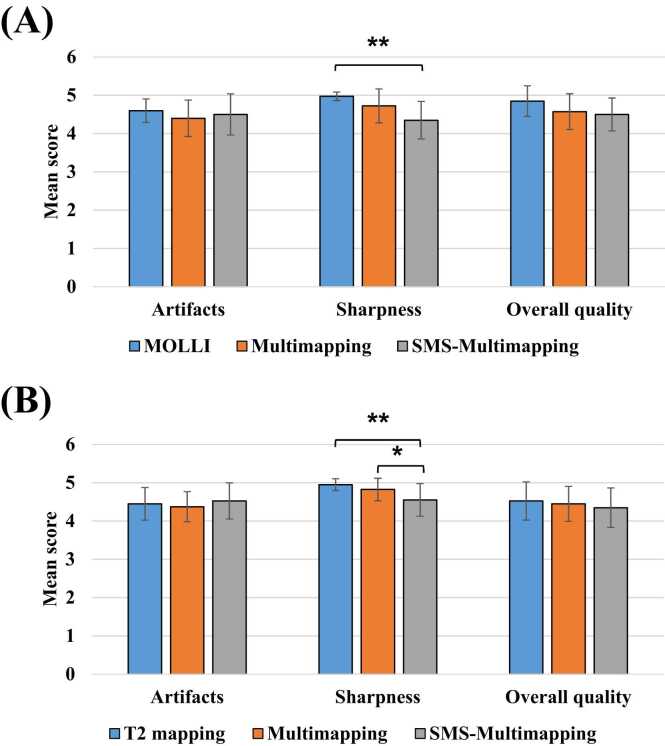
Qualitative comparison between MOLLI/T2 mapping, multimapping, and SMS-Multimapping (MB = 3) on 3 short-axis slices for 20 subjects, on a scale of 1 (worst) to 5 (best). The symbol */** represents P < 0.05/0.01. *MOLLI* modified look-locker inversion recovery, *MB* multiband, *SMS* simultaneous multislice

Fig. 10 shows the qualitative comparison between MOLLI/T2 mapping, multimapping, and SMS-Multimapping (MB = 3) on 3 short-axis slices for 20 subjects, on a scale of 1 (worst) to 5 (best). Three methods had significant differences in sharpness (P < 0.01 for T1 sharpness and P < 0.01 for T2 sharpness). For T1, SMS-Multimapping scored significantly lower than MOLLI (4.35 ± 0.48 vs 4.97 ± 0.11, P < 0.01); however, SMS-Multimapping scored similar to multimapping (4.72 ± 0.44, P = 0.15) on sharpness, and were not significantly different from MOLLI and multimapping on overall quality (4.5 ± 0.42 vs 4.85 ± 0.40 vs 4.58 ± 0.47, P = 0.054). For T2, SMS-Multimapping scored significantly lower than T2 mapping (4.55 ± 0.42 vs 4.95 ± 0.15, P < 0.01) and multimapping on sharpness (4.83 ± 0.29, P = 0.03); however, the overall quality score was not significantly different between T2 mapping, multimapping and SMS-Multimapping (4.53 ± 0.49 vs 4.45 ± 0.46 vs 4.35 ± 0.51, P = 0.11). There was no significant difference between three methods in terms of artifacts for both T1 (P = 0.71) and T2 (P = 0.12). ICCs of the two readers for the comparison were 0.61 (95% confidence interval [CI] [0.43, 0.73]) for artifacts, 0.71 (95% CI [0.58, 0.80]) for sharpness, and 0.75 (95% CI [0.65, 0.83]) for overall quality.

**Fig. 11:**
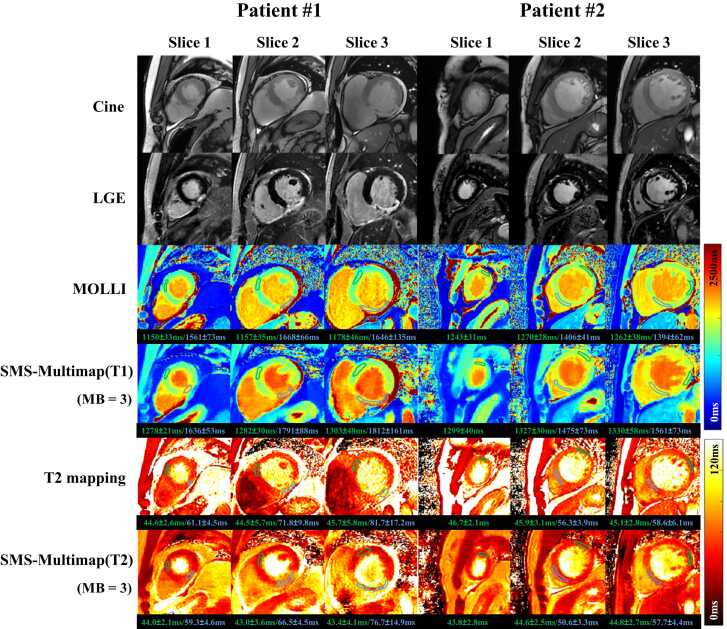
The T1, T2 maps, and slice-matched cines and LGEs in two patients: a 32-year-old male with an indication of myocardial infarction and a 41-year-old female with an indication of heart failure. T1 and T2 maps were obtained by MOLLI and T2 mapping (six breath-holds) and SMS-Multimapping for MB = 3 (one breath-hold). The T1 and T2 values (mean ± SD) in the diseased myocardium (blue contour) and remote myocardium (green contour) were noted below each map. *LGE* late gadolinium enhancement, *MOLLI* modified look-locker inversion recovery, *MB* multiband, *SMS* simultaneous multislice, *SD* standard deviation

Fig. 11 shows the T1, T2 maps obtained with routine methods and SMS-Multimapping, as well as the slice-matched cines and LGEs in two patients: a 32-year-old male with an indication of myocardial infarction and a 41-year-old female with an indication of heart failure. The quality of the SMS-Multimapping maps was overall comparable to that of reference methods despite using only one breath-hold for acquiring all the maps. ROIs were drawn in the diseased myocardium (blue) and remote myocardium (green). SMS-Multimapping showed similar trends of T1 and T2 changes between the diseased and remote myocardium compared with the reference methods. Fig. S2 shows the results for the other two patients. Overall, SMS-Multimapping showed good agreement with reference imaging methods in this preliminary patient evaluation.

## 4 Discussion

In this study, we propose a new framework for acquiring short-axis cardiac parametric maps, which comprises an SMS-Multimapping sequence and an LLR and sparsity-constrained reconstruction algorithm. The method is able to acquire myocardial T1 and T2 maps in maximally three slices in one breath-hold, achieving a total of six-fold accelerations. The LLRS reconstruction algorithm improves the quality of the raw images and maps relative to conventional reconstruction algorithms and exhibits good accuracy, reasonable precision, and acceptable reproducibility in a cohort of 20 healthy subjects. The potential of the proposed method to detect pathological T1 and T2 changes has been preliminarily demonstrated in four patients. The quality of the resultant T1 and T2 maps from the proposed approach was comparable to that of the conventional methods, despite using only one breath-hold to acquire all the data. Although a slight reduction of image sharpness is still found with SMS-Multimapping, our results support the potential of the proposed framework in achieving large accelerations of cardiac parameter mapping and improved patient comfort.

Several recent studies have reported the combination of SMS excitation and bSSFP readouts for cardiac imaging applications [19], [20], [21]. While bSSFP offers an improved SNR and is the mainstream method for single-band imaging, we used the FLASH readout in our method due to an integrated consideration regarding image artifacts, specific absorption rate (SAR), bias caused by field inhomogeneity, and total signal-to-noise ratio (SNR) of the reconstructed images. First, previous methods for combining bSSFP with SMS need to either change the original 0-π phase cycling used by bSSFP [21] or add gradient blips to the slice-selective gradient [36] to fulfill CAIPIRINHA encoding. However, both methods have reported increased artifacts. The first approach causes more banding artifacts in the heart due to the change of bSSFP phase-cycling scheme [21]. The second approach results in so-called positive banding artifacts, arising from the hyperenhanced stopband signals in the side-lobe excitations, which may significantly degrade the image quality as reported in a recent study on SMS cardiac T1 mapping [20]. Therefore, mitigation of these artifacts in SMS-bSSFP is a nontrivial task, whereas SMS-FLASH does not have these concerns. Second, SMS-bSSFP has considerably increased SAR compared with SMS-FLASH. The increased SAR not only restricts the highest MB factor allowed in SMS-bSSFP at 3T [11], but also in some cases requires an increase of TR, which further increases banding artifacts [36]. To date, the two published bSSFP-based cardiac SMS-T1 mapping studies were both at 1.5T [19], [20], suggesting this may be a general issue for application of SMS-bSSFP at high fields. Third, bSSFP itself has a higher sensitivity to B0 and B1 variations than FLASH [37] and may influence parameter estimation accuracy [38]. In our study, it has been noticed that bSSFP-based MOLLI and multimapping resulted in longer T1 values in the apical slice than the other two slices, whereas FLASH-based SMS-Multimapping did not. This improved uniformity may be due to the improved robustness of FLASH readouts to B0 and B1 field variations. Last, regarding the SNR concern, notice that the SMS excitation itself also increases the image SNR. Therefore, a combination of SMS with FLASH has superior SNR to FLASH alone, which may explain why the measured T1 and T2 SDs were not significantly different between multimapping and SMS-Multimapping.

On the reconstruction side, our method faces a 3-fold undersampling along the through-plane dimension and a near 4-fold undersampling along the in-plane dimension, resulting in a total of 12-fold acceleration. We note that the four-fold in-plane undersampling is needed to ensure that the acquisition window in each heartbeat is less than 200 ms. The large acceleration causes a significantly increased g-factor and d-factor penalty [39], which is combatted with by incorporation of the LLRS constraints. Before this work, GRAPPA+SPSG was used in many SMS methods for reconstruction [12], [13], [14], [19], [22], [23] and generated adequate image quality under low acceleration rates. In our study, we also performed GRAPPA+SPSG reconstruction for two-fold in-plane acceleration (results not shown) and found acceptable image quality. However, at a high acceleration rate such as 12, GRAPPA+SPSG suffers from strong artifacts and noise amplification, whereas the proposed LLRS reconstruction did not due to leveraging of the spatiotemporal redundancy in the raw images. The effect of combining LLR and sparsity has been demonstrated previously for MRF [25], but not for SMS parameter mapping. Our results show that this strategy also works well in this application.

After the LLRS reconstruction, we used the measured septal B1 to account for the overall impact of B1 inhomogeneity in the heart, which is a step inherited from the original mutlimapping study [8]. It is well known, however, that B1 is quite nonuniform across the heart at 3T, raising concerns about the accuracy of T1 and T2 estimations using the proposed correction method. For example, a previous study based on bSSFP multimapping showed that the wrongly estimated B1 can lead to an averaged error of 14 ms for T1 and an averaged error of 10 ms for T2 [8]. However, notice that the proposed SMS-Multimapping used a flip angle of 5° for the excitation, which is substantially lower than that of bSSFP readout. The low flip angle considerably reduces the sensitivity of the proposed approach against B1 variation. In a retrospective (Table S1) experiment, we found that changing the B1 factor from 0.9 to 0.7 only caused an average change of T1 by 26 ms and an average change of T2 by 0.5 ms, whereas the same changes for bSSFP-based multimapping were 32 ms and 3.9 ms, respectively (Table S1). This result suggests that our method has a reasonable robustness against B1 inhomogeneity at 3T. Incorporation of a multiflip-angle regime such as that introduced in [38] to jointly estimate T1, T2, and B1 may be an alternative approach that can further increase the robustness of the method against B1 variation.

Our framework has several advantages compared with other frameworks for SMS multiparametric mapping. First, our method uses Cartesian undersampling with only 11 heartbeats for acquisition, whereas SMS-MRF uses spiral sampling with a breath-hold of 16 heartbeats and SMS-multitasking uses radial sampling with a free-breathing scan of 3 min. Compared with these alternative approaches, our method has a higher robustness against hardware imperfections, such as eddy currents and gradient delay [40], due to the use of Cartesian undersampling, and a shortened scan time, which improves patient comfort and reduces relevant motion artifacts. Second, on the reconstruction side, the generation of dictionary in multimapping is much easier and faster than SMS-MRF, which needs simulation of signal over, e.g., 1000 contrast points (10 min) for each slice [8], [15]. The reconstruction time of the LLRS algorithm is also shorter than the other two methods, which requires 47 min for SMS-MRF and 2-3 h for SMS-multitasking, rendering the proposed method more amenable to clinical translation.

Despite reducing the scan time, the proposed framework manifested a lower T1 precision than MOLLI, lower sharpness scores than MOLLI and bSSFP T2 mapping, and a lower overall image quality than MOLLI. We noted that the T1 and T2 regional SD and scan-rescan reproducibility for the proposed method were still in a reasonable range, and comparable to those of regular multimapping. The reduced sharpness of the proposed method is probably an adverse effect of regularization, due to the large acceleration rate. Previous studies using LLR have reported similar occurrences of blurring [24], [25], [41]. Recently, there has been an increasing number of works utilizing supervised deep learning to improve the reconstruction quality of magnetic resonance parameter mapping at high acceleration rates [42], [43]. However, the use of supervised methods for SMS-Multimapping is challenging due to the lack of ground truth reconstructions. The proposed framework may provide a starting point to obtain training labels for training of new learning-based cardiac SMS-Multimapping methods, which is highly warranted in our future investigation.

## 5 Limitations

Our study has limitations. First, the method was only validated in 20 healthy subjects and 4 patients. Further larger studies involving more patient data are needed to fully verify the feasibility of the method in the clinical setting. Second, we only validated the method in a 3T scanner. It is unclear how this method would perform at 1.5T, which has a lower SNR than 3T. Finally, our method is also limited by the relatively long reconstruction time (∼30 min). Future development of supervised learning reconstruction methods may resolve this issue and facilitate clinical translation of the technique.

## 6 Conclusions

In conclusion, we propose an SMS-Multimapping framework that comprises a novel sequence and an LLR and sparsity regularized reconstruction. Results from phantom experiments, simulation, and prospective in vivo experiments indicate that SMS-Multimapping has good accuracy, reasonable precision, and acceptable reproducibility. The proposed LLRS algorithm for SMS-Multimapping is superior to traditional SMS algorithms in terms of reconstruction quality. However, the sharpness of the proposed method is still inferior to that of traditional methods. Nevertheless, the results suggest that the proposed framework has the potential to reduce the original six separate breath-holds to one breath-hold, dramatically increasing the scan efficiency of cardiac parametric mapping.

## Funding

This work was partially supported by the 10.13039/501100001809National Natural Science Foundation of China (No. 62001288) and the Shanghai Science and Technology Commission (No. 22TS1400200).

## Author contributions

Yinyin Chen: Data curation, Formal analysis, Investigation, Writing—original draft, Writing—review and editing. Chenxi Hu: Writing—review and editing, Writing—original draft, Validation, Methodology, Funding acquisition, Formal analysis, Conceptualization. Hang Jin: Data curation, Supervision, Writing—original draft, Writing—review and editing. Hongfei Lu: Data curation, Investigation, Writing—original draft, Writing—review and editing. Yixin Emu: Writing—review and editing, Writing—original draft, Validation, Methodology, Formal analysis, Data curation, Conceptualization, Investigation. Zhuo Chen: Formal analysis, Data curation, Writing—original draft, Writing—review and editing. Jianmin Yuan: Supervision, Funding acquisition, Writing—original draft, Writing—review and editing. Juan Gao: Formal analysis, Data curation, Writing—original draft, Writing—review and editing.

## Ethics approval and consent

The study of the healthy subjects was approved by Shanghai Jiao Tong University Ethics Review Board, and all participants provided informed written consent. The patient study was approved by Zhongshan Hospital Ethics Committee of Fudan University and Shanghai Medical Imaging Institute, and all patients provided informed written consent.

## Consent for publication

We confirm that a written informed consent for publication was obtained from every subject involved in the study.

## Declaration of competing interests

Jianmin Yuan reports a relationship with Central Research Institute (UIH Group) that includes employment. The other authors declare that they have no known competing financial interests or personal relationships that could have appeared to influence the work reported in this paper.

## Footnotes

Appendix A Supplementary data associated with this article can be found in the online version at doi:10.1016/j.jocmr.2024.101125.
