# Supplementary material for: Simultaneous multislice cardiac multimapping based on locally low-rank and sparsity constraints
Source: J Cardiovasc Magn Reson. 2024 Nov 14;26(2):101125. doi: 10.1016/j.jocmr.2024.101125 (PMC11663759; doi:10.1016/j.jocmr.2024.101125)
Supplement: Supplementary file 1 — Supplementary material [file mmc1.docx]

**Additional file 1**


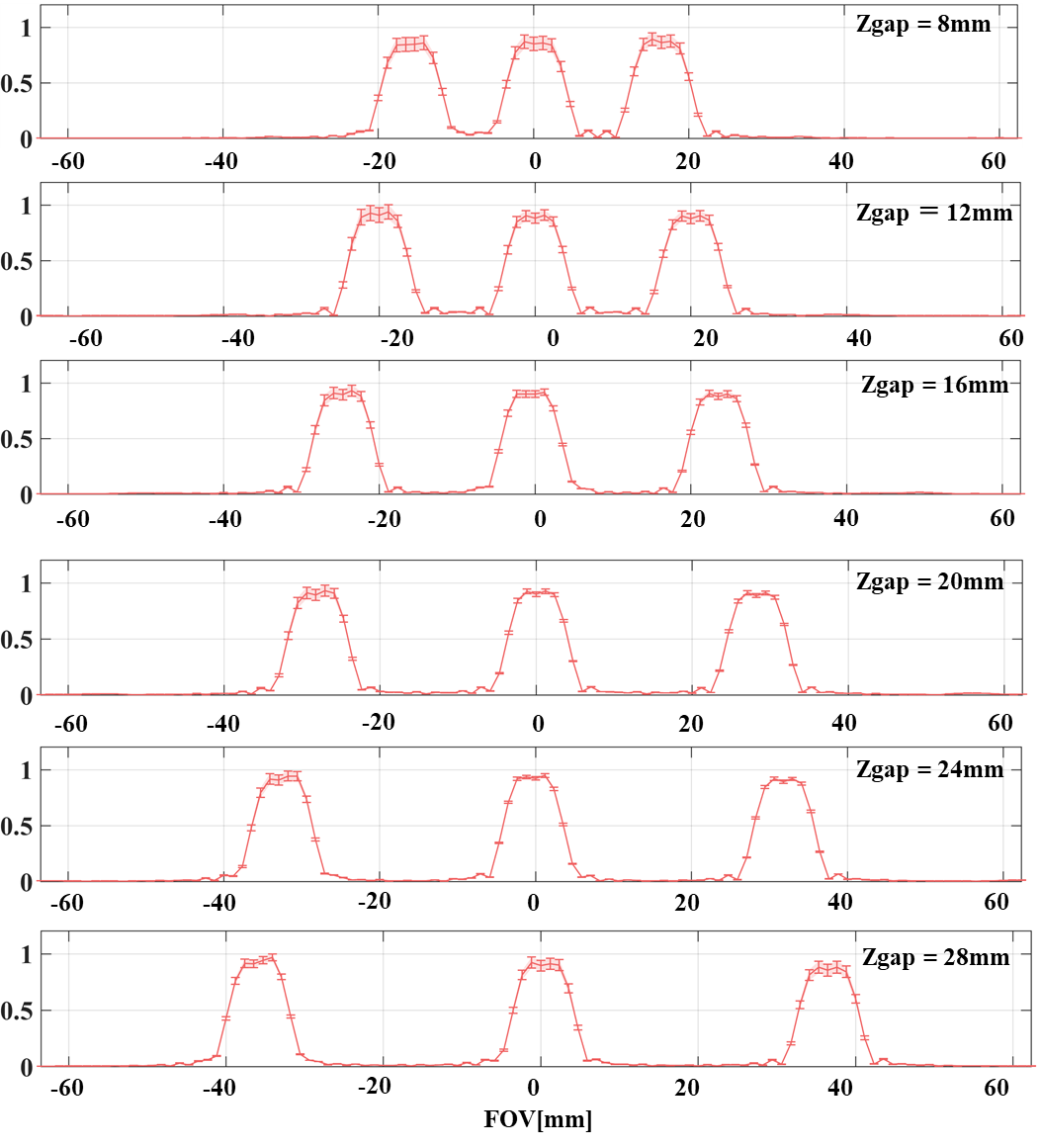


**Figure S1. Slice profile for different slice gaps (Zgap) of MB=3.** MB, multi-band.

**
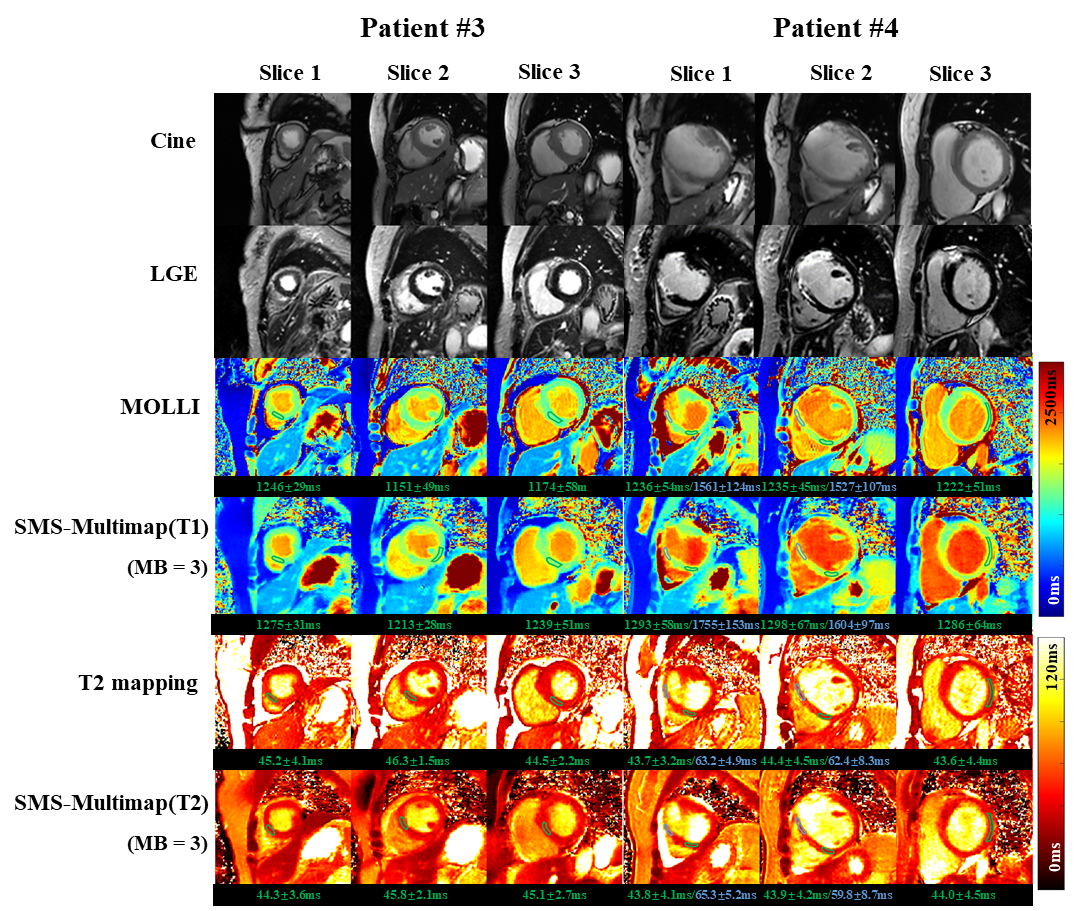
**

**Figure S2. The T1, T2 maps, and slice-matched Cines and LGEs in two patients: a 62-year-old female with an indication of hypertrophic cardiomyopathy; a 34-year-old female with an indication of myocarditis.** T1 and T2 maps were obtained by MOLLI and T2 mapping (6 breath-holds) and SMS-Multimapping for MB=3 (1 breath-hold). The T1 and T2 values (Mean±SD) in the diseased myocardium (blue contour) and remote myocardium (green contour) were noted below each map.

**Table S1. T1 and T2 values in the lateral wall (mean±SD) for different assumed B1+ factors in 10 healthy subjects (30 slices).**

|  | B1 factor | Multimapping | SMS-Multimapping |
| --- | --- | --- | --- |
| T1 | 0.7 | 1202±31ms | 1143±45ms |
|  | 0.9 | 1235±32ms | 1169±46ms |
| T2 | 0.7 | 40.7±2.8ms | 40.2±2.5ms |
|  | 0.9 | 44.6±3.4 | 40.7±2.5ms |
